# Editorial: Geopolymer and Alkali Activated Materials Chemistry, Structure, and Properties

**DOI:** 10.3389/fchem.2022.929163

**Published:** 2022-06-08

**Authors:** Cristina Leonelli, Kenneth John MacKenzie, Dong-Kyun Seo, Waltraud M. Kriven

**Affiliations:** ^1^ MacDiarmid Institute for Advanced Materials and Nanotechnology, Wellington, New Zealand; ^2^ School of Molecular Sciences, Arizona State University, Tempe, AZ, United States; ^3^ Department of Materials Science and Engineering, University of Illinois at Urbana-Champaign, Champaign, IL, United States

**Keywords:** geopolymers, alkali activated materials, mix design, characterization, foam, reinforcement, restoration, sustainability

This Research Topic gathers different contributions highlighting novel types of alkali activated materials and characterization methods to define the applications of these sustainable materials. These contributions allow us to shed light on the microstructure/performance relationship with focus on sustainability and process parameters.

The first article of this Topic (Ali Khan et al.) introduces three artificial intelligence (AI) techniques namely: artificial neural network (ANN), adaptive neuro-fuzzy interface (ANFIS), and gene expression programming (GEP) to establish a reliable and accurate model to estimate the compressive strength of fly-ash based geopolymer concrete. All three models also fulfil the external verification criterion suggested in the literature. Of particular interest is the proposed GEP equation that can be used in the preliminary design of fly-ash based geopolymer concrete.

The formulation of alkali activated materials from local Moroccan clays and sands is discussed by El Khomsi et al., who propose these materials as coatings for the restoration of historical monuments in Morocco. The authors reported that characterization of the substrates reveals differences in terms of pH value, capillarity, contact angle, and surface roughness. These differences affect the coating thickness, which also depends on the viscosity, liquid to solid ratio and granular skeleton of the geopolymer coating. High adhesive strength values (up to 9 MPa) were obtained on limestone, despite a strength decrease recorded with increasing relative humidity.

To produce metakaolin-based geopolymers for controlling humidity and permeability, the porosity of the geopolymer was determined by Petlitckaia et al. using the 3D X-ray tomography. The porosity, pore size distribution and constriction between adjacent cells, as well as the connection rates between pores were analyzed by the iMorph program. The results show that the total porosity increases from 26 to 74% when the initial concentration of the pore-forming H_2_O_2_ increases, which is in complete agreement with the tomography results. The effect of surfactants was also investigated.

Very often the porosity or cracks produced during drying of alkali activated binders must be avoided. A study to provide guidance regarding the choice of the optimal curing conditions to minimize deformations in slag-based alkali-activated materials was proposed by Češnovar et al.. The method was based on the modified ASTM C1698-19 standard for the measurement of autogenous shrinkage in cement pastes. Autogenous deformation and strain was measured in four samples using the standard procedure at room temperature, 40 and 60°C. The results show that the highest rate of autogenous shrinkage occurred at a temperature of 60°C, followed by drying shrinkage at 60°C and 30% relative humidity due to the fact that the rate of evaporation was highest at this moisture content.

Continuing with the problem of brittleness and poor crack resistance in alkali activated materials, the study by Meng et al. reported the mechanical properties of geopolymers with single-doped PVA fibres, single-doped carbon nanotubes, and mixed PVA fibers and carbon nanotubes. It was found that PVA fibres and carbon nanotubes exerted a positive improvement on the mechanical properties of the geopolymers, especially their bending strength and flexural strength. It was also found that the strengthening effect of PVA fibre on the geopolymer was primarily a physical strengthening effect, whereas the strengthening effect of carbon nanotubes on the geopolymers was both chemical and physical.

Still on the subject of characterizing the matrix/reinforcement interface, the contribution of Alzeer et al. reports on AAMs prepared from basalt glass having high compressive strengths (up to 90 MPa after 7 days of hydration) compared with those made using granulated blast furnace slag (GBFS). In addition, their calorimetry data show that the hydrolysis of the glass and the subsequent polymerization of the reaction product occur at a faster rate compared to GBFS-based samples, the presence of the glass formed sodium aluminosilicate hydrate (N-A-S-H) intermixed with Ca aluminosilicate hydrate gel (C-A-S-H), whereas alkali activation of GBFS produced predominantly C-A-S-H gel.

On the subject of microstructural models and their description, the review of Palomo et al., analyses the role of alkaline activators in the chemistry of alkali-activated binders (AABs). An important point is highlighted, namely, that alkaline activators are not by any means confined to the two synthetic products (caustic soda and waterglass) mostly employed by researchers and other sustainable and efficient products are widely available. Additionally, this review reports on the real potential of AABs as alternatives to Portland cement, the versatility of their production processes, the low carbon footprint of one-part AABs, and the urgent need to explore standardization formulas, thereby facilitating the commercial development of sustainable alternative binders to PC.

The alkali-activation reaction of metakaolin is a fairly complex process involving transformation of one amorphous reactant, metakaolin, into another amorphous product or products, N-A-S-H gel and/or disordered zeolite type phases. Abdelrahman and Garg address the relationship between the alkalinity of the mix and the extent of reaction. Applied in tandem, isothermal calorimetry, FTIR, XRD, TGA, NMR, and Raman imaging, reveal a clear but non-linear relationship between the Na/Al ratio and the extent of the alkali-activation reaction, indicating diminishing returns at higher Na/Al ratios, where higher Na/Al ratios cause an increase in the degree of reaction up to a certain point, after which increasing the Na/Al ratio does not significantly affect the reaction kinetics but may affect the gel polymerization.

We hope that the reader will find in this Research Topic much new and useful information in the field of geopolymers and alkali activated binders and materials.

